# Efficient and Fast Removal of Aqueous Tungstate by an Iron-Based LDH Delaminated in L-Asparagine

**DOI:** 10.3390/ijerph19127280

**Published:** 2022-06-14

**Authors:** Yaowu Cao, Qinghai Guo, Weihao Sun, Georgii A. Chelnokov

**Affiliations:** 1 State Key Laboratory of Biogeology and Environmental Geology, School of Environmental Studies, China University of Geosciences, Wuhan 430074, China; caoyaowu0105@126.com (Y.C.); tfyhjhiu@gmail.com (W.S.); 2 Faculty of Resources and Environmental Sciences, Hubei University, Wuhan 430062, China; 3 Geological Institute, Russian Academy of Sciences, 119017 Moscow, Russia; geowater@mail.ru

**Keywords:** tungstate removal, LDH, delamination, structural restoration

## Abstract

High concentrations of tungstate in aqueous systems pose a severe threat to the environment and human health. This study explored the potential of iron-based LDHs to remove tungstate from water. To improve its tungstate uptake capacity, environment-friendly L-asparagine was used to delaminate iron-based LDH synthesized via a coprecipitation method. The successful delamination was proved by AFM, revealing that the thickness of the obtained nanoparticles was approximately 1–2 times that of a single LDH layer. XRD, TEM, and XPS analyses confirmed that the delaminated LDHs were amorphous and ultrathin and had surface defects within their nanosheets that acted as active sites, leading to a very fast tungstate sorption rate and superior tungstate uptake capacity. Notably, the original layered structure of the L-asparagine-treated LDH was recovered upon its reaction with tungstate-bearing solutions, and therefore, the high availability of aqueous tungstate to the interlayer regions during the structural restoration of the delaminated iron-based LDH contributed to its excellent capability of tungstate removal as well. In addition, the tungstate uptake by the delaminated iron-based LDH was not affected substantially by the presence of coexisting anions, implying that the strong inner-sphere complexation between the tungstate and LDH layers with defects (i.e., Fe-O bonds) was the primary mechanism responsible for the tungstate removal. The delamination process described in this paper was validated to be an effective way to enhance the immobilization of tungstate by iron-based LDHs without inducing secondary pollutions, and delaminated iron-based LDHs are promising to be used extensively in the practice of treating tungstate-rich waters.

## 1. Introduction

Tungsten (W) has been widely used in industrial products that require high heat resistance and mechanical hardness, including lighting applications, electronic components, penetrators, and many metal alloys [1]. When tungsten exists in the form of metal or alloy, its toxicity is limited [1]. However, tungsten generally appears in its oxidized state (+VI valence) in natural waters, including monotungstates, polytungstates, and thiotungstates. Occurring in the form of various tungstates greatly improves the mobility of tungsten as well as its potential toxicity to plants, animals, and humans, which has been paid increasing attention in recent years [2,3,4,5]. Notably, environmental geochemical surveys since the early 2000s have indicated that the abnormally high tungstate concentrations of the groundwaters in Nevada (USA) up to 742 μg/L might be closely related to childhood leukemia [6,7,8,9]. Later, Kelly et al. [10] found that high levels of tungstate could cause tumor formation and DNA damage in wild-type mice. Some clinical studies have also shown that tungstate accumulation in human blood could cause cramps, stroke, or cardiovascular disease [11,12]. Therefore, tungsten in natural waters, especially those used as a drinking water source, should not exceed a certain upper limit, although there has been, so far, no drinking water standard for tungsten in most countries. Nevertheless, it has been listed as an emerging contaminant by some organizations, e.g., the United States Environmental Protection Agency (EPA).

Generally, the background concentrations of tungsten in natural waters are low. The average tungsten concentrations in atmospheric precipitation, river, and seawater are all less than 0.1 μg/L [13,14,15]. However, under the influence of certain human activities (e.g., tungsten mining and smelting, utilization of tungsten in military industries, discharge of industrial sewage) or natural processes (e.g., volcanic activity, weathering/leaching of tungsten-rich rocks), tungsten concentrations in natural waters may far exceed their background values [16]. For example, the geothermal springs in the Daggyai hydrothermal area (China) contain up to 961.2 μg/L of tungsten [17]; a maximum tungsten concentration of 0.56 mg/L was yielded in the groundwaters of Camp Edwards at the Massachusetts Military Reservation, USA [18]. In view of its detrimental effects on surrounding water resources, direct discharge of untreated tungsten-containing wastewaters should be prohibited.

Sorption is considered an extensively employed technique for the removal of toxic pollutants from water [19,20,21,22]. However, not much information is available about tungsten removal from aqueous solutions through sorption. The sorbents for the removal of tungsten that have been described in the literature include biosorbent [23,24], biopolymer-coated clay [25], carbon cloth [26], fly ash [27], boehmite [28], nanocrystalline iowaite [29], and carbon nanotubes [30]. Of them, iron-bearing sorbents have been given priority in view of the fact that iron is able to effectively complex with tungstate species. For example, garlic peel loaded with Fe(III) possessed a tungsten sorption capacity of 91.5 mg/g, much higher than that of garlic peel with either Te(IV) or Ce(III) being loaded [24]. Ogata et al. [31] also observed that a higher amount of tungsten was adsorbed onto the zeolite fly ash surface than onto the fly ash surface.

Layered double hydroxides (LDHs), also known as anionic clays, are a group of promising sorbents for tungstate uptake. LDHs can be described by a general formula [M^2+^_1−x_M^3+^_x_(OH)_2_][A^n−^_x/n_·mH_2_O], where M^2+^ and M^3+^ are di- and trivalent metal cations within the host layers of hydroxide sheets, and An- are interlayer anions. A positive charge on the host layers is derived from the isomorphous substitution of a fraction of divalent cations with trivalent cations and is compensated by the interlayer anions. Lamellar structure and anion exchange capability are prominent characteristics of LDHs [32,33,34,35]. Compared to bulky LDH solids, delaminated LDH nanosheets or positively charged two-dimensional ultrathin films would greatly improve the accessibility to the inner surfaces of host layers and extend the physical and chemical properties of anisotropic nanosheets [36,37]. Accordingly, it was expected that delaminated LDH nanosheets would have significant potential in sorbing aqueous anions. However, due to the high charge density of the host layers of LDH and the hydrogen bonding network among interlayer ions and water molecules, as well as LDH layers, the efficient and complete delamination of LDHs remains challenging. In recent years, the delamination of LDHs was directly achieved with the aid of the judicious selection of solvents [38,39]. For example, both NO_3_^−^-intercalated LDH (LDH-NO_3_) and CO_3_^2−^-intercalated LDH (LDH-CO_3_) could be delaminated in formamide [38,40,41], while the latter required an extra intensification process, including multi-step ultrasonic treatments or integrating ultrasonication and shaking [38] due to the stronger affinity of CO_3_^2−^ to LDH layers. Since formamide is difficult to recover after delamination due to its high boiling point (210 °C), alternatively, Shu et al. [42] delaminated 3D LDH-Cl into 2D nanosheets in acetone, which effectively accelerated boron removal from aqueous solutions. However, taking into account that both formamide and acetone are hazardous, more and more researchers advocated the use of environmental-friendly ways to delaminate LDHs [43,44]. A preferred non-hazardous solvent capable of delaminating LDHs is L-asparagine, a water-soluble amino acid whose carbonyl groups and amino groups have a strong interaction with LDH host layers and weaken their associations [36]. However, most works in this regard have been limited to delaminating Al-based LDHs [38,39,41,42,45]. In contrast, Fe(III) is more stable than Al(III) as the main cation in LDH layers, provoking a big challenge in the delamination of iron-based LDHs by L-asparagine.

In this study, we delaminated an iron-based LDH in L-asparagine and employed the delaminated LDH to remove tungstate from an aqueous solution for the first time. The major objectives were to characterize the composition, structure, and properties of the delaminated iron-based LDH samples before and after reacting with tungstate-bearing solutions to evaluate the capacities of delaminated iron-based LDH in terms of tungstate removal under different conditions via changing the contact time, initial tungstate concentration, solution pH, and coexisting anions, and to elaborate the mechanisms involved in tungstate removal from aqueous solutions. This study not only provides an environmentally friendly method for delaminating iron-based LDHs but also proposes a promising way to remove tungstate from water.

## 2. Materials and Methods

### 2.1. Synthesis of Iron-Based LDH

All chemical reagents were purchased from Sinopharm Chemical Reagent Co., Ltd. (Shanghai, China). Considering carbonate derived from CO_2_ in the air would be preferentially inserted into the interlayers and have a high affinity for LDHs, the deionized water involved in the preparation and washing was decarbonated prior to being boiled and subsequently cooled in N_2_ atmosphere for at least 30 min to remove the CO_2_ from the deionized water.

An LDH with a Mg/Fe ratio of 3 and nitrate intercalated (iron-based LDH) was synthesized using fast precipitation followed by a hydrothermal treatment method in accordance with our previous study [29]. Briefly, a mixed salt solution containing Mg(NO_3_)_2_ and Fe(NO_3_)_3_ was promptly added to the NaOH solution with vigorous stirring. The resulting brown LDH products were isolated by repeated centrifuging and the replacement of the supernatant water for further treatment. As a control experiment, the non-delaminated iron-based LDH (noted as L0) that was separated from the slurries before delamination was freeze-dried for analysis.

### 2.2. Delamination of Iron-Based LDH

After the centrifuging and washing process, the iron-based LDH sludges were dispersed into a saturated L-asparagine solution, which was prepared by adding 20 g of L-asparagine (i.e., 2,4-diamino-4-oxobutanoic acid, H_2_NCOCH_2_CH(NH_2_)COOH) into a conical beaker containing 400 mL of water at 45 °C for 20 min. Then the mixed suspension was sealed with N_2_ and underwent a continuous ultrasonic treatment in an ultrasonic bath for 30 min. Subsequently, the suspension was shaken at a speed of 200 rpm for 2 days for delamination. The formed colloidal suspension was further centrifugated at 4000 rpm for 10 min. After cooling, the stable homogeneous products (noted as L1) were further freeze-dried to avoid aggregation. The mass balance of the delaminated iron-based LDH during the preparation is shown in Table S1. The recoveries of Mg and Fe in the delaminated iron-based LDH after freeze-drying were 89.71% and 90.18%, respectively, which can be considered reasonable recovery rates.

### 2.3. Characterizations of the Synthesized Samples

The X-ray diffraction (XRD) patterns of solid products and reacted samples were performed by an X’Pert PRO DY2198 diffractometer with Cu Kα radiation of 0.8 mA and 45 kV, respectively. The spectra were collected with a step size of 0.02° and a speed of 5°/min in the 2θ range of 3 to 70°. FT-IR spectra of samples were recorded on a Bruker Vertex 70 spectrophotometer to characterize the functional groups of synthesized products. The morphologies of the samples were obtained by field-emission scanning electron microscope (FE-SEM, Hitachi Su-8010) and transmission electron microscopy (TEM, JEOL JEM-2100F). The surface of the sample was coated with Pt prior to SEM testing to increase the electrical conductivity of the samples and obtain clear scan images. Atomic force microscopy (AFM) images were obtained on a Bruker Dimension ICON platform. The suspension of samples was dropped on the conductive gel and naturally air-dried for SEM inspection, while the solid samples were dispersed in ethanol after 20 min of sonication for TEM and AFM detection. The Brunauer–Emmett–Teller (BET) surface areas of the products were obtained by nitrogen adsorption analysis using a Micromeritics ASAP2460. The surface compositions of the samples were identified by an XPS spectroscope (Thermo Scientific K-Alpha+, Waltham, MA, USA).

### 2.4. Sorption Tests

Sorption experiments were conducted in batch conditions by the delaminated iron-based LDH nanoparticles. The simulated tungsten solutions were prepared with a fresh stock solution of sodium tungstate (Na_2_WO_4_). Feed solutions (50 mL) were mixed with 0.1 g of sorbent and shaken in capped 100 mL polypropylene bottles. After the sorption, the resulting suspensions were centrifuged, and the supernatants were filtered for later chemical analyses. Duplicate tests were run to ensure the data quality.

For sorption kinetics, the initial tungstate concentration was fixed at 5 mM (25 °C), and samples were collected at various reaction times. Sorption isotherms at various temperatures (25, 45, and 65 °C) were performed using simulated tungstate solutions, with initial concentrations ranging from 0.0001 to 5 mM. Furthermore, 0.1 mM tungstate solutions mixed with 0.2/0.5/1 mM of Cl^−^, SO_4_^2−^, HCO_3_^−^, and PO_4_^3−^ were included in batch sorption experiments to assess the influence of competitive anions on tungstate removal. Batch experiments were also performed with 0.1 mM tungstate solutions with a wide pH range of 2 to 12, which was adjusted by a small amount of dilute HCl or NaOH solutions and preequilibrated for 72 h to ensure the stable existence of multiple tungstate species under different pH conditions. The pH values of all solutions were measured by a Mettler Toledo pH meter. The concentrations of tungsten in the filtrates were determined by inductively coupled plasma mass spectrometry (ICP-MS) (iCAP, Thermo Fish) in view of its high sensitivity and low detection limits for analyzing metal and metalloid elements [46,47,48,49].

### 2.5. Sorption Model Fitting

The experimental kinetic data were fitted using the pseudo-first-order (PFO) and pseudo-second-order (PSO) models, as shown in Equations (1) and (2), respectively:

(1) $$\ln\left(q_{e}-q_{t}\right)={\mathrm{lnq}}_{e}-k_{1}t$$

(2) $$\frac{t}{q_{t}}=\frac{t}{q_{e}}+\frac{1}{k_{2}q_{e}^{2}}$$

where q_t_ and q_e_ (mg/g) are the amounts of adsorbate sorbed at time t (min) and equilibrium, respectively, and K_1_ (min^−1^) and K_2_ (g·mg/min) are the rate constants of the PFO and PSO models, respectively.

The adsorption data were processed by the Langmuir, Freundlich, and Sips isotherm models, described by Equations (3)–(5) [50]:

(3) $$\frac{c_{e}}{q_{e}}=\frac{1}{K_{L}\cdot q_{m}}+\frac{c_{e}}{q_{m}}$$

(4) $$q_{e}=K_{F}c_{e}^{1/n}$$

(5) $$q_{e}=q_{m}K_{s}c_{e}^{\frac{1}{m}}/\left(1+K_{s}c_{e}^{\frac{1}{m}}\right)$$

where c_e_ (mg/L) is the equilibrium tungsten concentration in the aqueous solution, q_m_ (mg/g) is the maximum sorption capacity, K_L_ is defined as the Langmuir constant, which is related to the free energy of sorption, K_F_ is the Freundlich constant, K_s_ is the Sips constant related to the energy of sorption, and parameters n and m are indicative of the sorption intensity and system heterogeneity, respectively.

## 3. Results

### 3.1. Characteristics of Synthesized Sorbent

**Figure 1:**
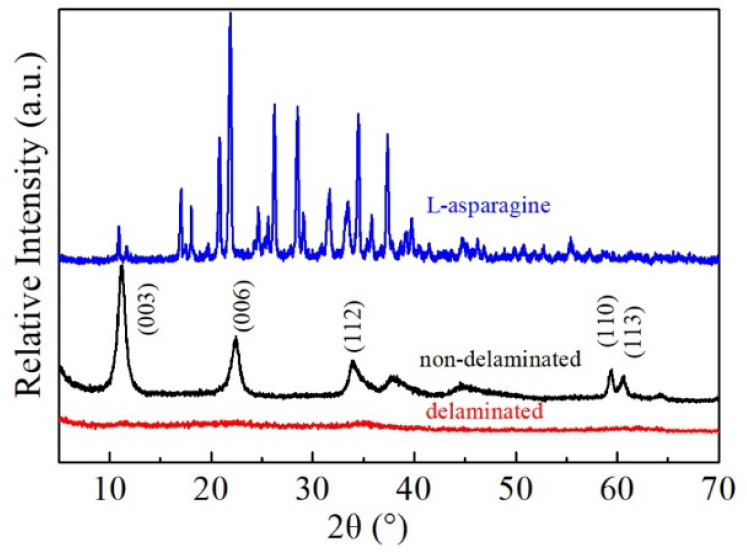
XRD patterns of L-asparagine, non-delaminated, and delaminated iron-based LDH.

The XRD patterns of L-asparagine, original, and delaminated iron-based LDH are shown in Figure 1. The XRD pattern of the original sample (L0) reveals that it is highly crystallized, with similar XRD lines as previously reported in the literature [51,52,53]. The strong reflections observed at the 2θ values of 11.2°, 22.4°, 34.0°, 59.4°and 60.6° were typical for the (003), (006), (012), (110), and (113) diffraction planes, corresponding to a rhombohedral structure of LDH [54,55]. Among these planes, the (003) reflection indicates a basal reflection with d-spacing of 0.788 nm, which is the theoretical layer spacing of the original LDH sample with intercalated nitrate. In contrast, after delamination in L-asparagine, the XRD pattern of the iron-based LDH product (L1) features a flat curve, similar to the diffraction characteristic of amorphous minerals, where the sharpness and intensity of the reflections disappeared dramatically and only a weak and broad reflection was observed in the 2θ range of 30–40°. This indicates that the crystallinity of the iron-based LDH decreased and the delamination of the iron-based LDH in L-asparagine did occur.

**Figure 2:**
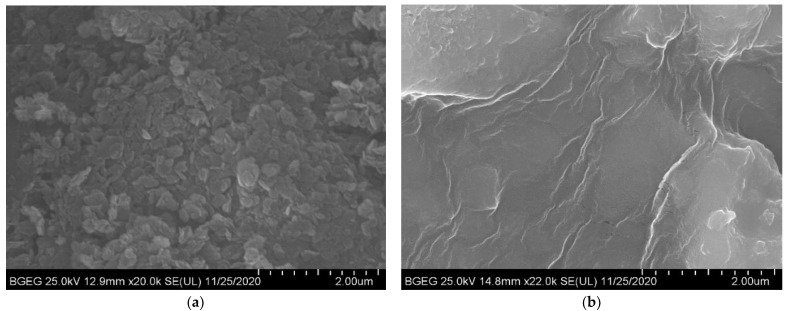
SEM images of non-delaminated (**a**) and delaminated iron-based LDH (**b**).

**Figure 3:**
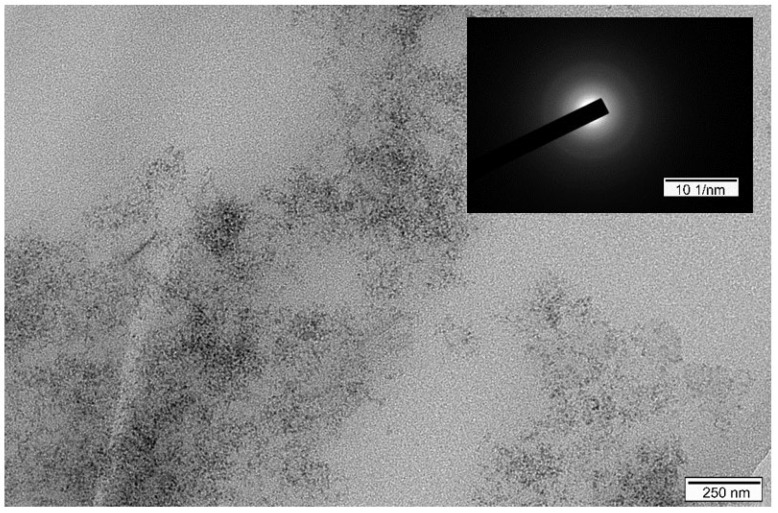
TEM image of delaminated iron-based LDH.

The morphology of sample L1 was depicted using SEM and TEM, and the corresponding images are shown in Figure 2 and Figure 3, respectively. Prior to the delamination, the commonly acknowledged crystalline structures of the as-prepared L0 and its agglomerated plate-like particles were clearly observed (Figure 2a). As expected, after the delamination in L-asparagine solution, the crystalline structure of the sample disappeared, smooth surfaces formed, and there were no plate-like particles at 22,000 magnification, which corresponded well with the XRD results. This was most likely caused by the L-asparagine, whose C=O groups strongly interacted with the LDH layers to form new weak hydrogen bonds, depleting a high number of interlayer water molecules and leading to the structural collapse of the original LDH, along with the well stack of the amorphous LDH nanoparticles during freeze-drying. Nevertheless, in the process of drying, the products were assembled by continuous LDH layers so that the pinholes and defects were not visible, showing a highly smooth membrane surface (Figure 2b). Likewise, the TEM image of the L1 sample shows that it was composed of transparent, ultrathin nanosheets with a size of less than 10 nm (Figure 3). Moreover, the selected area electron diffraction (SAED) pattern of the sample indicates its amorphous nature. It can be speculated that when stirred into L-asparagine, the LDH layers had a strong interaction with the carbonyl groups of L-asparagine, which replaced the original interlayer water molecules. Thus, the integrated hydrogen bonding network was broken, producing a loosely stacked and highly swollen phase, which was expected to improve the accessibility to the inner surfaces of the host layers. In the process of breaking the hydrogen bonding network, some hydrogen-bonded hydroxyl groups would also be broken and become metastable, and then run off to form coordinatively unsaturated metal centers, i.e., defect-rich structures, as demonstrated by [56,57,58]. These surface defects within the ultrathin nanosheets would create much more active sites and increase the positive charges of the intrinsic surfaces, which could be beneficial to the sorption of the target components, especially anionic contaminants. Indeed, the N_2_ adsorption/desorption measurements used to study the surface properties of the original and delaminated iron-based LDH samples show that both of them exhibited a type IV isotherm, indicating the existence of micropores and mesopores (Figure S1). The delaminated iron-based LDH sample had a much larger specific surface area (199 m^2^/g) compared to the original LDH sample (92 m^2^/g).

**Figure 4:**
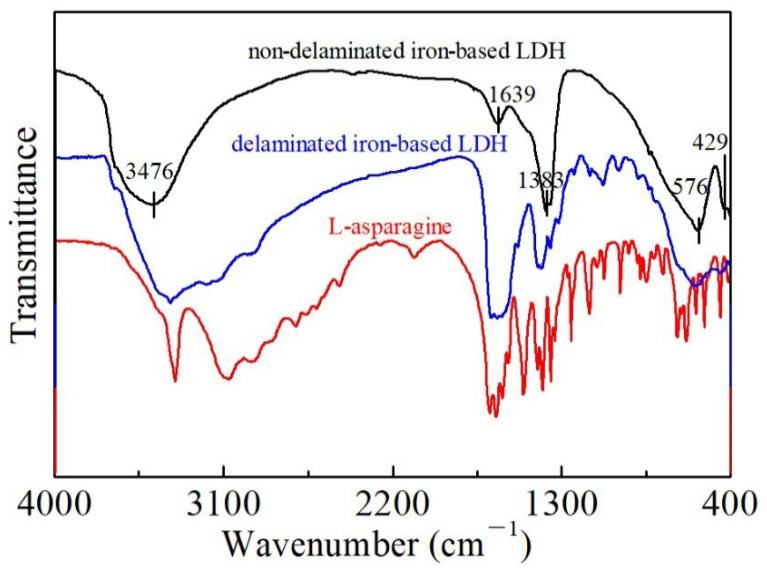
FT-IR spectra of the non-delaminated and delaminated iron-based LDH samples.

Furthermore, the intercalation of L-asparagine into the delaminated iron-based LDH was also evidenced by its FT-IR spectra (Figure 4). In the FT-IR spectra of the non-delaminated L0 sample, a sharp peak at 1383 cm^−1^ was observed and attributed to the N-O stretching vibration mode of NO_3_^−^. The broad and strong band at 3476 cm^−1^, the weak band at 1639 cm^−1^, and the bands at 576 and 429 cm^−1^ were attributed to the O-H stretching modes, the H_2_O bending vibration, and the metal-oxygen (M-O) stretching and bending modes, respectively. In contrast, due to the substitution of L-asparagine for NO_3_^−^ and water molecules in the interlayers, the vibration band of the delaminated LDH at 1383 cm^−1^ was weakened, and new peaks at 1750–850 cm^−1^, indicative of the existence of C-H, C-C, and C=O, were produced, which can be ascribed to the νas (COO-) mode. To sum up, FT-IR spectra along with the XRD, SEM, and TEM results revealed that L-asparagine molecules were indeed in the interlayers of the L1 samples, which are basically amorphous nanoparticles.

**Figure 5:**
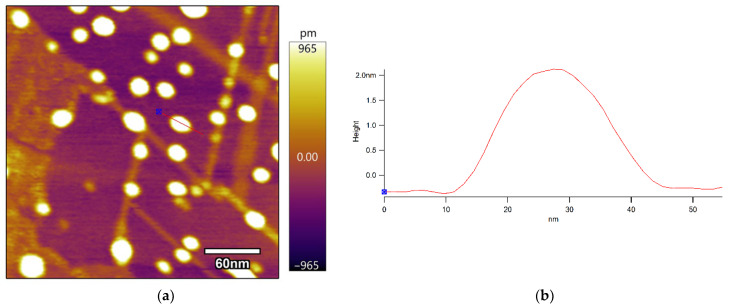
AFM image (**a**) of delaminated iron-based LDH and the height profile (**b**) of nanoparticles corresponding to the red line in the AFM image.

Finally, atomic force microscopy was used to determine the accurate size of a delaminated iron-based LDH nanoparticle. Figure 5 reveals that the LDH nanoparticles had a uniform lateral size of 20–30 nm. The height profile scan indicates that the thickness of the nanoparticles was approximately 2 nm. In view of the fact that the height of a typical LDH monolayer suggested by the relevant literature is 0.8–1 nm [40,41], which is up to the sum of the crystallographic thickness of an LDH basal layer (0.48 nm) and the diameter of an intercalated anion or L-asparagine molecule, the obtained delaminated iron-based LDH should have no more than two monolayers in its structure.

### 3.2. Sorption Kinetics

**Figure 6:**
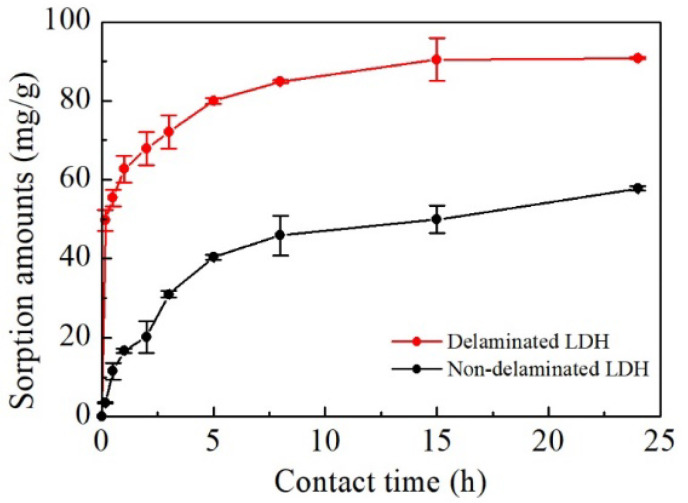
Reaction rates of tungstate sorption by delaminated and non-delaminated iron-based LDH.

The kinetic curves in Figure 6 indicate that the delaminated iron-based LDH performed better tungsten sorption than the non-delaminated iron-based LDH under the same conditions, with a faster sorption rate especially in the early stage of the experiments. For the delaminated iron-based LDH, the sorption amount of tungstate increased sharply in a very short time (<30 min) and leveled off after around 3 h due to the rapid decrease in active sorption sites. After 15 h, the tungstate sorption by the delaminated iron-based LDH was at full equilibrium. Faster sorption rates are preferable for the practical application of delaminated iron-based LDH in the treatment of tungstate-bearing waters.

Pseudo-first-order (PFO) and pseudo-second-order (PSO) kinetic models were employed to evaluate the kinetic sorption processes of tungstate to the delaminated and non-delaminated iron-based LDH (Figure S2). The correlation coefficient for the PSO model (R^2^ > 0.980) was higher than that for the PFO model (R^2^ > 0.910), implying that chemical sorption via sharing or exchanging electrons between the sorbents and the sorbed tungstate was involved in the sorption experiments, as suggested by [59]. Based on the PSO model, the sorbed tungstate concentration at equilibrium (q_e_) and the sorption rate constant (k_2_) of both the delaminated and non-delaminated iron-based LDH at different initial tungstate concentrations were calculated and are listed in Table 1. Notably, the calculated q_e_ and k_2_ of the delaminated iron-based LDH were 92.2 mg/g and 0.0236, respectively, and much higher than those of the non-delaminated iron-based LDH (60.7 mg/g and 0.0067), indicating a great improvement in both the sorption capacity and sorption rate after the delamination treatment.

### 3.3. Sorption Isotherms

**Figure 7:**
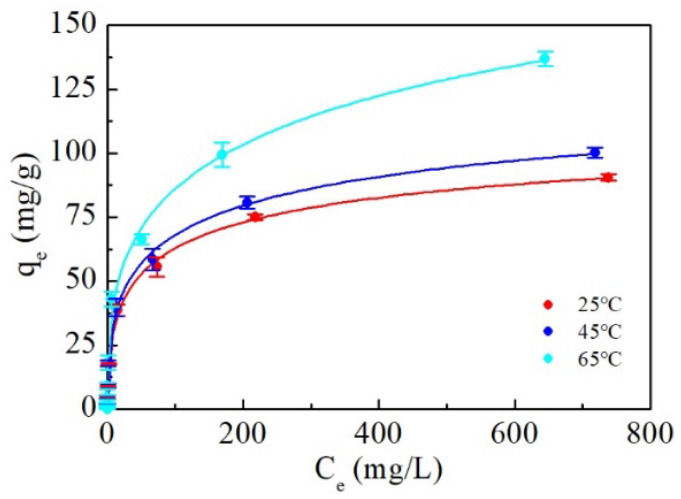
Isotherms of tungstate sorbed onto delaminated iron-based LDH and their fits to Sips model.

Table S1 recorded the sorption isotherm data of the delaminated iron-based LDH at 25, 45, and 65 °C, respectively. It is clear that the sorption of tungstate to the delaminated iron-based LDH significantly increased with the increasing temperature, indicating that a high temperature was beneficial for the tungstate removal. Furthermore, the sorption data were fitted by the Langmuir, Freundlich, and Sip isotherm models to evaluate the tungstate sorption capacity. Sorption isotherms are depicted in Figure 7 and Figure S3, and Table 2 shows the isotherm parameters along with the R^2^ values. The experimental equilibrium data for tungsten sorption adapted a little better to the Sips model, whose correlation coefficient was higher than 0.99. However, the differences among the fits to the three models were very small; that is, the Langmuir and Freundlich models satisfactorily fitted the sorption data equally well (R^2^ > 0.98). Hence, both the monolayer combination and multilayer sorption mechanisms suggested by these sorption models were involved in the tungstate sorption to delaminated iron-based LDH. A reasonable explanation here is that the Sips isotherm model is capable of covering a wide range of tungstate concentrations. In other words, the Sips isotherm model is a combined form of the Langmuir and Freundlich expressions. It is plausible to speculate that the Freundlich isotherm model is more applicable at high tungstate concentrations, while at low tungstate concentrations, monolayer sorption, indicated by the Langmuir isotherm model, is the predominant process.

The tungsten sorption performance of the delaminated iron-based LDH was compared with those of other sorbents reported in the literature and is presented in Table 3. The experimental sorption capacity of the delaminated iron-based LDH under the same condition was 90.5 mg/g at room temperature, which is much better than most of the other sorbents listed in Table 3, including a non-delaminated iron-based LDH that had a maximum sorption capacity of 58.4 mg/g under the same conditions (Table S3). Therefore, it is safe to conclude that delaminated iron-based LDH is a promising sorbent for practical tungsten removal from aqueous solutions.

### 3.4. Effect of pH on Tungstate Removal

The solution pH value is one of the most critical parameters that affect a sorption process and it has been extensively studied for the sorption of oxyanions, such as arsenate and arsenite. Tungsten forms various oxyanions under different pH values, including monooxyanions and polyoxyanions [1,61], which makes the effects of pH on its removal more complicated.

**Figure 8:**
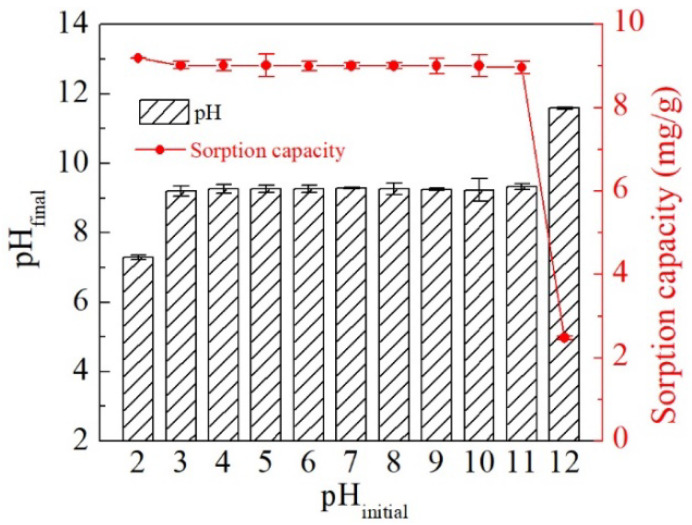
Effect of initial pH on tungstate sorption by delaminated iron-based LDH with reaction conditions: initial tungsten concentration = 0.1 mM, solid/solution ratio = 2 g/L, reaction time = 24 h, and T = 25 °C.

Nevertheless, the delaminated iron-based LDH still exhibited good stability for tungstate removal over a range of pH values. Specifically, at the initial tungsten concentration of 0.1 mM, its tungstate sorption capacity was about 9 mg/g at pH values ranging from 3 to 11, while it was slightly higher than 9 mg/g at pH 2 and significantly lower than 9 mg/g at pH 12 (Figure 8). This indicates that the sorption reaction was little affected by the pH under acidic, neutral, and weakly-alkaline conditions. As shown in Figure S4, tungstate tends to form polymeric species or exist in different valence states in acid solutions. However, by adding delaminated iron-based LDH to tungstate solutions with initial pH values of 3 to 11, the final solution pH values were all adjusted to about 9.20, which was attributed to the buffer effect of the LDHs [62]. In such cases, the solution tungstates were sorbed in the form of monomeric WO_4_^2−^ to delaminated iron-based LDH. In contrast, when the initial solution pH was 2, the final pH was correspondingly much lower, at which a small number of polymeric tungstates were expected to form. Therefore, the tungstate removal efficiency was improved at the initial pH of 2 probably because polymeric tungstates are somewhat liable to be sorbed by delaminated iron-based LDH than monomeric tungstates. Despite the 10-fold increase in the initial tungstate concentration, the sorption capacity remained stable in the narrow range of 50.91–50.82 mg/g at a pH of 4–10 (Table S4), which is consistent with the performance at a tungstate concentration of 0.1 mmol/L. By increasing the initial solution pH to 12, the final solution pH changed just a little. Hence, the excess OH- ions in the solution and their competitive sorption with WO_4_^2−^ resulted in a significant decrease in tungsten sorption by the delaminated iron-based LDH.

### 3.5. Effect of Co-Existing Ions on Tungstate Removal

**Figure 9:**
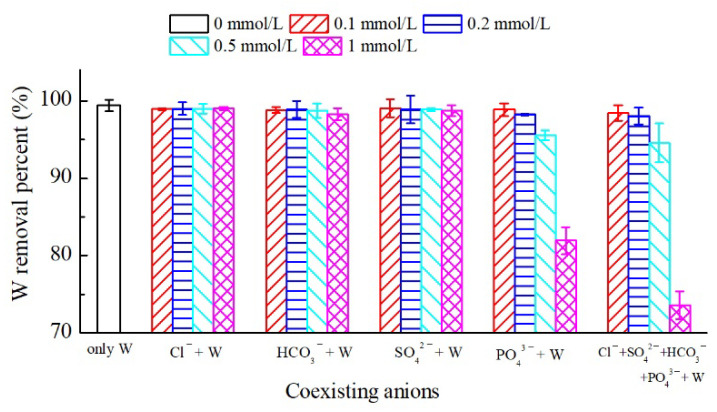
Effects of co-existing anions on tungstate sorption by delaminated iron-based LDH with reaction conditions: initial tungsten concentration = 0.1 mM, solid/solution ratio = 2 g/L, reaction time = 24 h, and T = 25 °C.

In natural waters or wastewaters, oxyanions (such as HCO_3_^−^, SO_4_^2−^, and PO_4_^3−^) and chloride ions are widely present. These coexisting anions increase the ionic strength of the solution and are apt to compete with tungstate ions for the surface sorption sites of delaminated iron-based LDH. Therefore, with the coexistence of the above-mentioned anions, the selective sorption efficiency of delaminated iron-based LDH to tungsten was studied (Figure 9). When the chloride concentrations ranged from 0 to 1 mmol/L, the tungstate removal efficiency showed little change, while tungstate removal was somewhat inhibited at HCO_3_^−^ and SO_4_^2−^ concentrations up to 1 mmol/L. As for PO_4_^3−^ ions, they could suppress tungstate removal a little at high concentrations as well. The tungstate removal percentage decreased from 99.5% without PO_4_^3−^ ions being added to 95.6% and 82.0% at 0.5 and 1 mmol/L PO_4_^3−^, respectively. Nevertheless, the tungstate removal percentage kept above 70% regardless of the initial concentrations of Cl^−^, HCO_3_^−^, SO_4_^2−^, and PO_4_^3−^, which was superior to the non-delaminated iron-based LDH (50–60% [29]). Hence, the delamination of the non-delaminated iron-based LDH not only improved its tungstate removal efficiency but also made its selective tungstate sorption performance much better.

The effects of coexisting anions on tungstate removal were also further studied by increasing the tungstate concentration 10-fold to 1 mmol/L without any unexpected results. As shown in Table S5, the overall effect of competing anions remained consistent with the strength of the anion exchange capacity, which is in agreement with the initial tungstate concentration of 0.1 mmol/L. Tungstate removal was somewhat inhibited at Cl-, HCO_3_^−^, and SO_4_^2−^ concentrations of 1 mmol/L, while it was suppressed greatly when PO_4_^3−^ anions were added to the tungstate solution due to the strong electronegativity.

### 3.6. Characterization of the Solid Phases after Sorption

**Figure 10:**
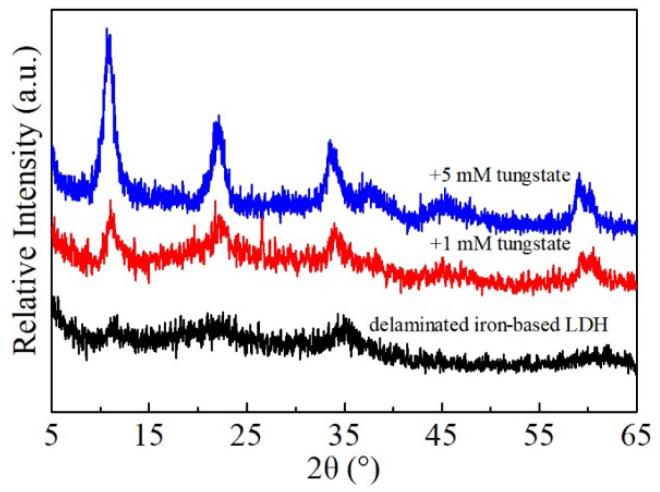
XRD patterns of delaminated iron-based LDH after reacting with tungstate-bearing solutions.

After reaction with tungstate-bearing solutions, the solid phases were sampled and subsequently characterized by a combination of XRD, SEM, TEM, FT-IR, and XPS analyses. The XRD patterns of the reacted delaminated iron-based LDHs showed seven diffraction peaks (Figure 10) similar to those of the non-delaminated iron-based LDH, implying that tungstate, instead of negatively charged L-asparagine molecules in the solution, could act as intercalated anions and was substantially involved in the structural reconstruction of amorphous iron-based LDH nanoparticles. Indeed, with the increase in the initial tungsten concentration in the solution, the XRD peaks of the recrystallized LDHs became sharper. However, the reacted non-delaminated LDH products still had seven XRD diffraction peaks (Figure S5). This indicates that no new minerals were formed, and the tungstate sorption by the non-delaminated LDHs was mainly attributed to the anion exchange between the interlayer nitrate and tungstate in the solution. Hence, the higher tungsten uptake by the delaminated iron-based LDHs was definitely related to the greater availability of tungstate to the interlayer regions in view of the fact that the interlayers were formed via their reactions with tungstate solutions. Instead, for non-delaminated iron-based LDH, the interlayers were originally existent and, therefore, tungstate must diffuse from the solution into the interlayers to replace the original intercalated anions balancing the positive charges of the layers.

**Figure 11:**
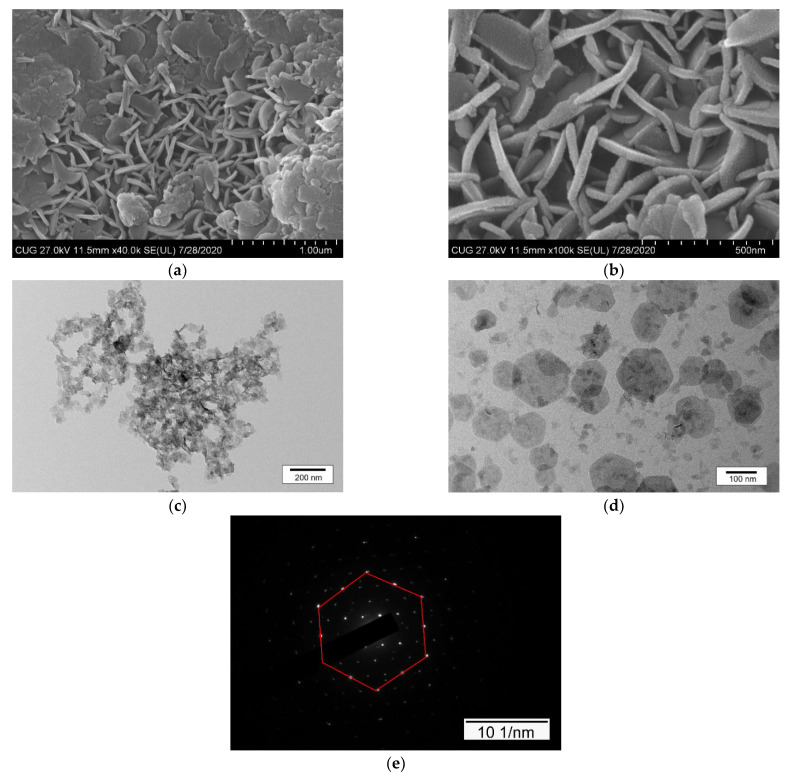
The SEM images (**a**,**b**) of delaminated iron-based LDH after tungstate sorption showing crystalline morphology and TEM (**c**,**d**) and the corresponding SAED pattern (**e**).

The SEM and TEM results also suggest the reconstruction of non-delaminated iron-based LDH in tungstate-bearing solutions. As shown in Figure 11, the crystalline plate-like particles appeared and the pores were filled and arranged irregularly upon tungstate sorption, which was quite different from the flat surface of the unreacted delaminated iron-based LDH. The lateral sizes of the plates were approximately 200–500 nm and their thickness was about 30–40 nm, equivalent to 10 times the size of delaminated iron-based LDH. TEM analysis revealed that the stable suspensions of the reacted samples were dispersed particles with a well-shaped hexagonal form (Figure 11c,d). The SAED pattern of an individual nanosheet showed hexagonally arranged spots (Figure 11e), confirming its single-crystal nature and the reconstruction of the crystalline layered structure as well.

**Figure 12:**
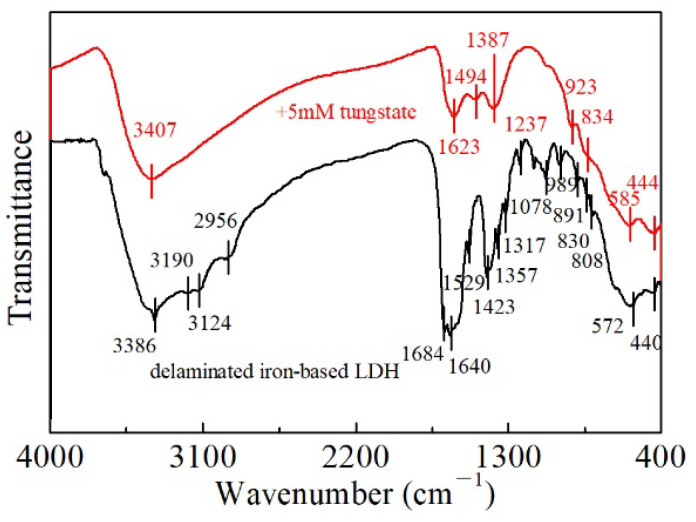
FT-IR spectra of delaminated iron-based LDH before and after reaction with 5 mM tungstate.

As discussed above, tungstate entered the interlayer regions and restored the layered structure of the amorphous iron-based LDH nanoparticles in the process of its rehydration. However, the competitive sorption studies indicated that the coexisting anions with stronger electronegativity and smaller ion radii, such as SO_4_^2−^ and PO_4_^3−^, did not completely replace the sorbed tungstate, even if their initial concentrations were 10 times higher than that of tungstate. Hence, the surface complex was likely the major mechanism responsible for the tungstate sorption. Indeed, it was also observed in the FT-IR spectra of the reacted solid samples (Figure 12) that the typical peaks of -COO- (808–1684 cm^−1^) of delaminated iron-based LDH disappeared and the vibrations of Fe(Mg)-O at 440–572 cm^−1^ slightly shifted to higher wavenumbers, once again indicating the important role of metal/oxygen bonds in tungstate sorption.

**Figure 13:**
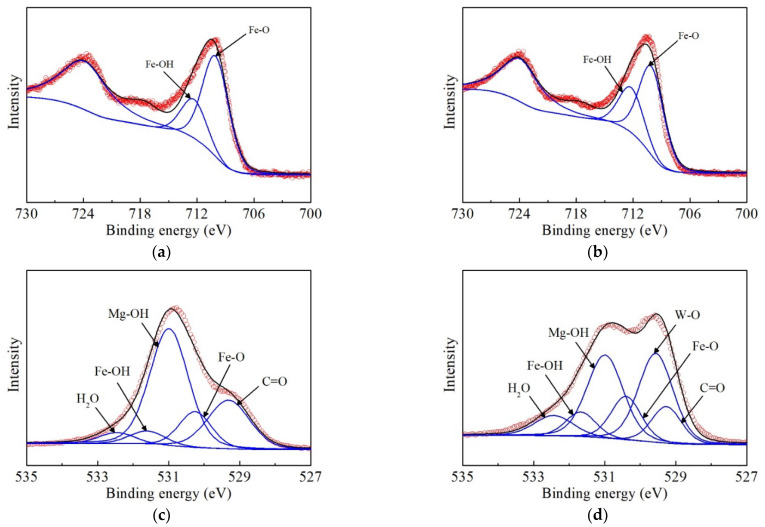
XPS spectra of Fe 2p for delaminated iron-based LDH before (**a**) and after the sorption (**b**), and the narrow scan of O 1s before (**c**) and after the sorption (**d**).

Evidence for the occurrence of surface tungstate complex came from the XPS analyses as well (Figure 13, Figures S6 and S7). The main elements in delaminated iron-based LDH are Mg, Fe, N, C, and O. Tungstate removal by delaminated iron-based LDH seems unrelated to the Mg atoms in its structure in view of the slim difference in the Mg 2p spectra of the solid samples before and after the tungstate sorption (Figure S6b). The N 1s spectra of the non-delaminated iron-based LDH and L-asparagine were used to calibrate the standard binding energies of the nitrate and amide groups. As shown in Figure S7b, for the non-delaminated LDH, only the characteristic peak of nitrate (406.83 eV) was displayed in the N 1s spectra, while the characteristic peak of the amide groups appeared at a lower binding energy (398–402 eV). In view of this, the N 1s spectra of the delaminated LDH before and after tungstate sorption were analyzed again. The characteristic peak of the nitrate groups was barely visible for the delaminated LDH, while it showed a sharp peak of amide groups at 399.34 eV, indicating that L-asparagine, instead of nitrate, did enter the interlayers during the preparation and delamination of iron-based LDH. However, after tungstate sorption, the binding energy of N 1s spectra was almost not changed, while its peak intensity reduced significantly, indicating that amide groups in delaminated iron-based LDH derived from L-asparagine could be replaced by aqueous tungstate to a high degree during its rehydration. The characteristic peaks of W 4f, with binding energies of 34.98 and 37.03 eV, were detected in the reacted samples (Figure S6d). Compared to the reported binding energies of various tungstate compounds (Ng and Hercules, 1976; Nefedov et al., 1982; Chowdari et al., 1992), these two peaks were attributed to the chemical bonds of WO_4_^2−^ in W 4f7/2 and W 4f5/2, respectively. This confirms that aqueous tungstate species were eventually fixed on the surface of the delaminated iron-based LDH via chemical sorption.

Unlike the Mg, N, and C spectra, the Fe atoms in the delaminated iron-based LDH had significant changes in both the binding energy and chemical composition upon tungstate sorption. The proportion of Fe atoms bonded to OH (written as Fe-OH) in the total Fe atoms in the unreacted delaminated iron-based LDH was calculated to be 28.2%, while that of Fe atoms bonded to O (written as Fe-O) was 71.8% (Figure 13a and Table 4). This is quite different from conventional LDHs synthesized via coprecipitation that contain mainly metal/hydroxyl bonds [29,63]. As a result, these excess Fe-O bonds in delaminated iron-based LDH formed a defect-rich structure, i.e., coordinatively unsaturated metal centers, which were the main active sites for inner-sphere tungstate complexation to form Fe-O-W bonds. In contrast, the proportion of Fe-OH in the reacted iron-based LDH increased to 34.1% (Table 4), suggesting that the delaminated LDH could restack upon its contact with tungstate solutions, as already validated by XRD, SEM, TEM, and FT-IR analyses. Similarly, the O 1s spectrum of the delaminated iron-based LDH, composed of five components at 529.3 eV (C=O), 530.3 eV (Fe-O), 531.0 eV (Mg-OH), 531.6 eV (Fe-OH), and 532.5 eV (H_2_O) (Figure 13c), changed significantly upon tungstate sorption. Specifically, due to the uptake of tungstate by the delaminated iron-based LDH, the ratio of the H_2_O peak area increased from 7.8% to 10.8%, while the ratio of the C=O peak area was reduced from 23.8% to 8.0%. Notably, a new signal at 529.6 eV was displayed in the O 1s spectrum of the reacted sample, which corresponds to the W-O bonds. Its peak area accounts for one-third of that of the O 1s spectrum, confirming the efficient tungstate uptake by delaminated iron-based LDH.

To summarize, the tungstate removal by the delaminated iron-based LDH was primarily controlled by inner-sphere complexation combined with anion exchange. The delaminated iron-based LDH exhibited fast reaction kinetics and excellent tungstate uptake, which was due to the adequate anion exchange of nanoparticles along with reconstruction in the tungstate solution. Meanwhile, the strong synergistic effect between tungstate and LDH with defects was responsible for the formation of the inner-sphere complexation, which was less influenced by common anions in the case of competitive sorption.

## 4. Conclusions

In this paper, an environment-friendly and effective route to the delamination of iron-based LDH in L-asparagine is provided. The carbonyl groups in L-asparagine had a strong interaction with the iron-based LDH layers to replace its interlayer water molecules, and as a result, the original crystalline iron-based LDH changed to an amorphous phase finally. The AFM results prove that the iron-based LDH was indeed delaminated to amorphous nanoparticles with a thickness of approximately 2 nm, equivalent to 1–2 times the thickness of a single LDH layer. The delaminated iron-based LDH was able to remove tungstate from water fast and efficiently. The tungstate sorption data were well fitted to the pseudo-second-order sorption model and the Sip isothermal model. The maximum tungsten sorption capacity of the delaminated iron-based LDH was calculated to be 90.5 mg/g, much higher than those of the non-delaminated iron-based LDH and most other reported sorbents for tungstate removal. Moreover, the delaminated iron-based LDH exhibited a relatively stable tungstate uptake ability over a wide range of pH values and with the coexistence of common anions, such as Cl^−^, HCO_3_^−^, SO_4_^2−^, and PO_4_^3−^. The superior sorption performance of the delaminated iron-based LDH was attributed to the high availability of tungstate to the interlayer regions during its structural restoration and the formation of inner-sphere complexes with tungstate on its surface defects. These results suggest that delaminated iron-based LDH has a huge potential to be applied in the practice of removing excess tungstate in natural waters.

## Figures and Tables

**Table 1:** Kinetic model parameters for tungstate sorption onto delaminated iron-based LDH.

Sorbent	Lagergren First-Order Model			Pseudo Second-Order Model		
	k_1_	q_e, cal_	R^2^	k_2_	q_e, cal_	R^2^
Non-delaminated LDH	0.141	44.2	0.910	0.0067	60.7	0.980
Delaminated LDH	0.307	44.8	0.979	0.0236	92.2	0.999

**Table 2:** Isotherm parameters for tungstate sorption on delaminated iron-based LDH.

	Langmuir Isotherm			Freundlich Isotherm			Sips Isotherm			
	q_m,L_	K_L_	R^2^	n	K_F_	R^2^	q_m,S_	K_S_	m	R^2^
25 °C	90.9	0.0821	0.993	3.64	15.8	0.980	126	0.117	2.16	0.998
45 °C	100	0.0943	0.992	3.61	17.2	0.983	152	0.109	2.29	0.997
65 °C	136	0.0856	0.985	3.42	21.3	0.991	326	0.0648	2.69	0.995

**Table 3:** Sorption capacities of various sorbents for tungsten.

Sorbent	Sorbent Dosage (g/L)	pH	q_m_ (mg/g)	References
Fly ash-based zeolites	2.5	2–3	62.34	[27]
Ag nanoparticle-treated activated carbon	1	4	20.15	[26]
Biopolymer coated clay/natural clay			23.9/5.45	[25]
Fe-sepiolite		3.0	54.6	[60]
Multi-walled carbon nanotubes	0.5	6	25.18	[30]
Fe(III) modified garlic peel	2	2.5	91.5	[24]
Nanocrystalline iowaite	2	2–12	71.9	[29]
Non-delaminated iron-based LDH (25 °C)	2	2–12	57.3	This study
Delaminated iron-based LDH (25 °C)	2	2–12	90.5	This study

**Table 4:** Fitting parameters for Fe 2p3/2 and O 1s XPS spectra.

Sample	Peak	Fe 2p3/2		O 1s	
		Binding Energy (eV)	Percent (%)	Binding Energy (eV)	Percent (%)
Delaminated iron-based LDH	Mg-OH			531.0	52.5
	Fe-OH	712.3	28.2	531.6	5.6
	Fe-O	710.0	71.8	530.3	13.5
	H_2_O			532.5	4.6
	C=O			529.3	23.8
After sorption	Mg-OH			531.0	30.0
	Fe-OH	712.3	34.1	531.6	7.5
	Fe-O	710.0	65.9	530.3	13.1
	W-O			529.6	30.6
	H_2_O			532.4	10.8
	C=O			529.3	8.0

## Data Availability

All data included in this study are available upon request by contacting the corresponding author.

## Supplementary Materials

The following supporting information can be downloaded at: https://www.mdpi.com/article/10.3390/ijerph19127280/s1, Figure S1: N2 adsorption/desorption isotherms of non-delaminated and delaminated iron-based LDH; Figure S2: The fitting plots of the pseudo-first-order model (a,c) and pseudo-second-order model (b,d) for delaminated and non-delaminated iron-based LDH; Figure S3: Isotherms of tungstate sorbed onto delaminated iron-based LDH and their fits to Langmuir model (a) and Freundlich model (b); Figure S4: Aqueous tungstate speciation at total tungstate concentrations of 0.1 mM calculated using the program PHREEQC at ionic strength 0.01 M; Figure S5: XRD patterns of non-delaminated iron-based LDH after reacting with tungstate-bearing solutions; Figure S6: XPS spectra of delaminated iron-based LDH. The wide scan of the amorphous LDH before and after sorption (a), the narrow scan of Mg 1s (b), C 1s (c), and W 4f (d); Figure S7: XPS spectra of N 1s for delaminated iron-based LDH before and after sorption (a) and non-delaminated iron-based LDH and L-asparagine (b); Table S1: The mass balance of delaminated iron-based LDH during the preparation; Table S2: Experimental isotherm data of tungsten removal by delaminated iron-based LDH; Table S3: Experimental isotherm data of tungsten removal by non-delaminated iron-based LDH at 25 °C; Table S4: Effects of pH on tungstate removal by delaminated iron-based LDH at an initial tungsten concentration of 1mM; Table S5: Effects of co-existing anions on tungstate removal by delaminated iron-based LDH at an initial tungsten concentration of 1 mM.
