# Supplementary material for: Efficient and Fast Removal of Aqueous Tungstate by an Iron-Based LDH Delaminated in L-Asparagine
Source: Int J Environ Res Public Health. 2022 Jun 14;19(12):7280. doi: 10.3390/ijerph19127280 (PMC9223674; doi:10.3390/ijerph19127280)
Supplement: Supplementary file 1 [file ijerph-19-07280-s001.zip › Supplementary File/Figure S7.pdf]

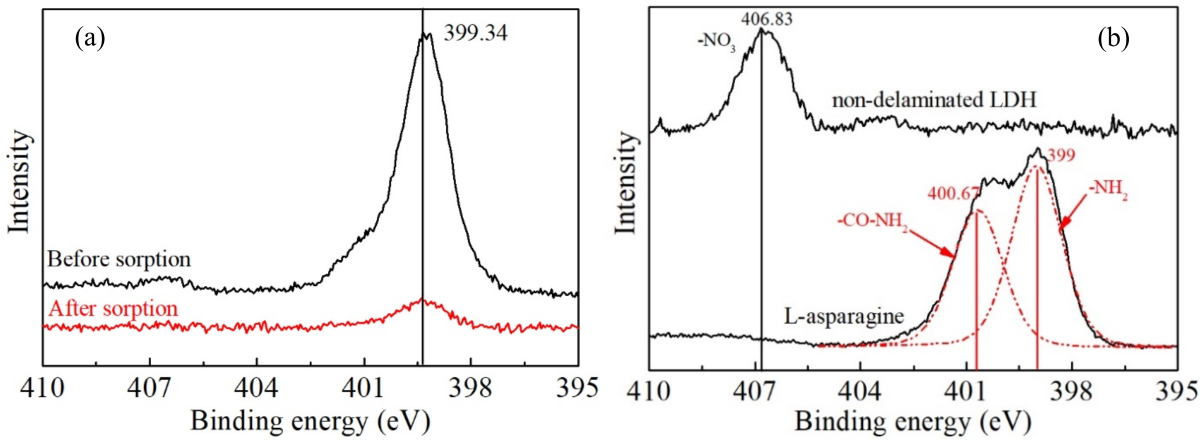

Figure S7. XPS spectra of N 1s for delaminated iron-based LDH before and after sorption (a) and non-delaminated iron-based LDH and L-asparagine (b).
