# Supplementary material for: Efficient and Fast Removal of Aqueous Tungstate by an Iron-Based LDH Delaminated in L-Asparagine
Source: Int J Environ Res Public Health. 2022 Jun 14;19(12):7280. doi: 10.3390/ijerph19127280 (PMC9223674; doi:10.3390/ijerph19127280)
Supplement: Supplementary file 1 [file ijerph-19-07280-s001.zip › Supplementary File/Table S1.pdf]

Table S1. The mass balance of delaminated iron-based LDH during the preparation.

Items	Values	Items	Values
Total mass of delaminated LDH after freeze drying	7.268 g		
Amount of Mg added for preparation	1.44 g	Amount of Fe added for preparation	1.12 g
Percentage of Mg content in delaminated LDH obtained by digestion	17.77%	Percentage of Fe content in delaminated LDH obtained by digestion	13.90%
Amount of Mg in delaminated LDH after freeze drying	1.292 g	Amount of Fe in delaminated LDH after freeze drying	1.010 g
Recovery of Mg in the preparation of delaminated LDH	89.71%	Recovery of Fe in the preparation of delaminated LDH	90.18%
