# Supplementary material for: Efficient and Fast Removal of Aqueous Tungstate by an Iron-Based LDH Delaminated in L-Asparagine
Source: Int J Environ Res Public Health. 2022 Jun 14;19(12):7280. doi: 10.3390/ijerph19127280 (PMC9223674; doi:10.3390/ijerph19127280)
Supplement: Supplementary file 1 [file ijerph-19-07280-s001.zip › Supplementary File/Table S2.pdf]

Table S2. Experimental isotherm data of tungsten removal by delaminated iron-based LDH.

25 °C				45 °C			
c_o (mg/L)	c_e (mg/L)	q_e (mg/g)	R (%)	c_o (mg/L)	c_e (mg/L)	q_e (mg/g)	R (%)
0.0184	0.002	0.008	89.7	0.0184	0.0007	0.009	96.2
0.0919	0.004	0.044	96.2	0.0919	0.001	0.045	99.0
0.184	0.003	0.090	98.4	0.184	0.001	0.091	99.2
0.919	0.008	0.455	99.1	0.919	0.007	0.456	99.2
1.84	0.017	0.911	99.1	1.84	0.014	0.912	99.3
3.68	0.032	1.82	99.1	3.68	0.038	1.82	99.0
9.19	0.108	4.54	98.8	9.19	0.095	4.55	99.0
18.4	0.353	9.01	98.1	18.4	0.397	8.99	97.8
36.8	1.77	17.5	95.2	36.8	0.943	17.9	97.4
91.9	14.7	38.6	84.0	91.9	12.4	39.7	86.5
184	72.7	55.5	60.4	184	71.8	56.0	61.0
368	218	75.0	40.8	368	184	91.7	49.9
919	738	90.5	19.7	919	719	100	21.8

65 °C			
c_o (mg/L)	c_e (mg/L)	q_e (mg/g)	R (%)
0.0184	0.0001	0.009	99.6
0.0919	0.0003	0.046	99.6
0.184	0.003	0.090	98.5
0.919	0.004	0.457	99.5
1.84	0.007	0.915	99.6
3.68	0.018	1.83	99.5
9.19	0.061	4.56	99.3
18.4	0.131	9.12	99.3
36.8	0.565	18.1	98.5
91.9	5.81	43.0	93.7
184	51.0	66.4	72.3
368	169	99.4	54.1
919	645	137	29.8
