# Supplementary material for: Efficient and Fast Removal of Aqueous Tungstate by an Iron-Based LDH Delaminated in L-Asparagine
Source: Int J Environ Res Public Health. 2022 Jun 14;19(12):7280. doi: 10.3390/ijerph19127280 (PMC9223674; doi:10.3390/ijerph19127280)
Supplement: Supplementary file 1 [file ijerph-19-07280-s001.zip › Supplementary File/Table S3.pdf]

Table S3. Experimental isotherm data of tungsten removal by non-delaminated iron-based LDH at 25℃.

Initial W concentration (mg/L)	Equilibrium W concentration (mg/L)	Removal percent (%)	Sorption capacity (mg/g)
1.838	0.0202	98.90	0.9089
9.190	0.1102	98.80	4.540
18.38	2.801	84.76	7.790
91.90	30.33	66.99	30.78
183.8	103.3	43.82	40.27
367.6	253.0	31.19	57.32
919.0	802.3	12.70	58.37
