# Supplementary material for: Efficient and Fast Removal of Aqueous Tungstate by an Iron-Based LDH Delaminated in L-Asparagine
Source: Int J Environ Res Public Health. 2022 Jun 14;19(12):7280. doi: 10.3390/ijerph19127280 (PMC9223674; doi:10.3390/ijerph19127280)
Supplement: Supplementary file 1 [file ijerph-19-07280-s001.zip › Supplementary File/Table S4.pdf]

Table S4. Effects of pH on tungstate removal by delaminated iron-based LDH at an initial tungsten concentration of 1mM.

Initial pH	Equilibrium pH	Initial W concentration (mg/L)	Equilibrium W concentration (mg/L)	Removal percent (%)	Sorption capacity (mg/g)
2	3.08	115.7	0.00903	99.99	57.85
4	8.89	183.8	78.46	57.31	52.67
6	9.13	183.8	80.24	56.34	51.78
8	9.12	183.8	78.17	57.47	52.82
10	9.15	183.8	81.97	55.40	50.91
12	11.54	183.8	142.6	22.40	20.58
