# Supplementary material for: Efficient and Fast Removal of Aqueous Tungstate by an Iron-Based LDH Delaminated in L-Asparagine
Source: Int J Environ Res Public Health. 2022 Jun 14;19(12):7280. doi: 10.3390/ijerph19127280 (PMC9223674; doi:10.3390/ijerph19127280)
Supplement: Supplementary file 1 [file ijerph-19-07280-s001.zip › Supplementary File/Table S5.pdf]

Table S5. Effects of co-existing anions on tungstate removal by delaminated iron-based LDH at an initial tungsten concentration of 1mM.

Co-existing anions	Cl^-/HCO_3^-/SO_4^{2-}/PO_4^{3-} concentration (mmol/L)	Initial W concentration (mmol/L)	Equilibrium W concentration (mmol/L)	Removal percent (%)	Sorption capacity (mg/g)
Only WO_4^{2-}	0	1	0.387	61.3	56.3
WO_4^{2-}+Cl^-	1	1	0.458	54.2	49.8
WO_4^{2-}+HCO_3^-	1	1	0.491	50.9	46.8
WO_4^{2-}+SO_4^{2-}	1	1	0.524	47.6	43.8
WO_4^{2-}+PO_4^{3-}	1	1	0.732	26. 8	24.6
WO_4^{2-}+Cl^-+HCO_3^-+SO_4^{2-}+PO_4^{3-}	4[a]	1	0.803	19.7	18.1

[a]: Cl^-, HCO_3^-, SO_4^{2-} and PO_4^{3-} coexisted in the solution at a concentration of 1 mmol/L for each anion, i.e., the total concentration of coexisting anions was 4 mmol/L except for tungstate.
